# Unbiased bioinformatic and clinical analysis identifies glial fibrillary acidic protein as a potential biomarker in spinal muscular atrophy

**DOI:** 10.1093/braincomms/fcag350

**Published:** 2026-09-11

**Authors:** Karina Lúcia Soares de Oliveira, Arthur Carpeggiani Weber, Michele Michelin Becker, Juliana Silva de Almeida Magalhães, Ana Letícia Amorim de Albuquerque, Júlia Kersting Chadanowicz, Giovanna Câmara Giudicelli, Francieli Rohden, Eduardo Rigon Zimmer, Thayne Woycinck Kowalski, Marina Siebert, Marcela Câmara Machado Costa, Jonas Alex Saute

**Affiliations:** Graduate Program in Medicine, Medical Sciences, Federal University of Rio Grande do Sul, Porto Alegre, RS 90035003, Brazil; Clinical Neurogenetics Research Group, Hospital de Clínicas de Porto Alegre, Porto Alegre, RS 90035903, Brazil; Clinical Neurogenetics Research Group, Hospital de Clínicas de Porto Alegre, Porto Alegre, RS 90035903, Brazil; Child Neurology Unit, Hospital de Clínicas de Porto Alegre, Porto Alegre, RS 90035903, Brazil; Neuromuscular Disease Center, Escola Bahiana de Medicina e Saúde Pública, Salvador, BA 40285001, Brazil; Graduate Program in Medicine, Medical Sciences, Federal University of Rio Grande do Sul, Porto Alegre, RS 90035003, Brazil; Clinical Neurogenetics Research Group, Hospital de Clínicas de Porto Alegre, Porto Alegre, RS 90035903, Brazil; Clinical Neurogenetics Research Group, Hospital de Clínicas de Porto Alegre, Porto Alegre, RS 90035903, Brazil; Bioinformatics Core, Hospital de Clínicas de Porto Alegre, Porto Alegre, RS 90035903, Brazil; Graduate Program in Genetics and Molecular Biology, Federal University of Rio Grande do Sul, Porto Alegre, RS 91501-970, Brazil; Graduate Program in Biological Sciences: Biochemistry, Universidade Federal do Rio Grande do Sul, Porto Alegre, RS 90035-003, Brazil; Graduate Program in Biological Sciences: Biochemistry, Universidade Federal do Rio Grande do Sul, Porto Alegre, RS 90035-003, Brazil; Graduate Program in Biological Sciences: Pharmacology and Therapeutics, Universidade Federal do Rio Grande do Sul, Porto Alegre, RS 90050170, Brazil; Department of Pharmacology, Federal University of Rio Grande do Sul, Porto Alegre, RS 90050170, Brazil; Bioinformatics Core, Hospital de Clínicas de Porto Alegre, Porto Alegre, RS 90035903, Brazil; Graduate Program in Genetics and Molecular Biology, Federal University of Rio Grande do Sul, Porto Alegre, RS 91501-970, Brazil; Medical Genetics Service, Hospital de Clínicas de Porto Alegre, Porto Alegre, RS 90035903, Brazil; Unit of Laboratorial Research, Experimental Research Center, Hospital de Clínicas de Porto Alegre (HCPA), Porto Alegre, RS 90035903, Brazil; Graduate Program in Gastroenterology and Hepatology, Federal University of Rio Grande do Sul, RS 90035003, Porto Alegre, Brazil; Neuromuscular Disease Center, Escola Bahiana de Medicina e Saúde Pública, Salvador, BA 40285001, Brazil; Graduate Program in Medicine, Medical Sciences, Federal University of Rio Grande do Sul, Porto Alegre, RS 90035003, Brazil; Clinical Neurogenetics Research Group, Hospital de Clínicas de Porto Alegre, Porto Alegre, RS 90035903, Brazil; Graduate Program in Genetics and Molecular Biology, Federal University of Rio Grande do Sul, Porto Alegre, RS 91501-970, Brazil; Medical Genetics Service, Hospital de Clínicas de Porto Alegre, Porto Alegre, RS 90035903, Brazil; Department of Internal Medicine, Federal University of Rio Grande do Sul, Porto Alegre, RS 90035003, Brazil

**Keywords:** spinal muscular atrophy, GFAP, neurofilament light chain, differential gene expression, biomarker

## Abstract

Spinal muscular atrophy (SMA) is an autosomal recessive disorder caused by bi-allelic pathogenic variants in *SMN1*, leading to motor neuron degeneration, muscle atrophy and progressive weakness. Although disease-modifying therapies have recently become available, reliable biomarkers for disease monitoring remain limited. This study aimed to identify potential biomarkers of disease severity and monitoring in SMA using an unbiased approach based on differential gene expression datasets. A meta-analysis of datasets from 15 studies in SMA mouse models identified genes differentially expressed in at least three studies. Of the 134 differentially expressed genes, 122 had human orthologs, with only two being CNS-specific and consistently altered across studies, including glial fibrillary acidic protein (GFAP), a marker of astrocyte reactivity. Candidate gene expression was subsequently evaluated in two human datasets. GFAP and neurofilament light chain (NfL) serum levels were then measured in a case–control study including 25 SMA patients (11 type 1, 14 types 2/3) and 24 matched controls, followed by a 12-month cohort study of the SMA patients. GFAP levels in cerebrospinal fluid (CSF) were assessed in 14 patients before and up to 14 months after initiation of disease-modifying therapies. Serum GFAP levels were lower in SMA patients compared with controls (*P* = 0.014), but higher in those with SMA type 1 (*P* = 0.028) and in patients with two *SMN2* copies (*P* = 0.03). Patients under disease-modifying treatment exhibited higher baseline GFAP levels (*P* = 0.01). CSF GFAP levels decreased after 14 months of treatment in SMA type 1 and in pre-symptomatic patients predicted to develop type 1 (*P* = 0.045). NfL levels were elevated in SMA patients, correlating with disease severity and *SMN2* genotype, and further increased over 12 months. Taken together, this study, employing an unbiased biomarker discovery strategy, identified GFAP as a candidate biomarker for SMA. Clinical validation demonstrated reduced serum GFAP levels, which correlated with disease type, *SMN2* copy number and treatment status. In SMA type 1, CSF GFAP levels declined over time with treatment. These findings implicate astrocytes in SMA-related neurodegeneration and support GFAP as a potential biomarker for the disease.

## Teaser

Spinal muscular atrophy lacks disease-monitoring biomarkers. Using meta-analysis of differential gene expression datasets and clinical validation, de Oliveira *et al*. report glial fibrillary acidic protein (GFAP) is a candidate biomarker. Serum GFAP correlates with disease type, *SMN2* copies and treatment status, while CSF GFAP decreases during therapy in type 1.

## Graphical Abstract

**Graphical Abstract:**
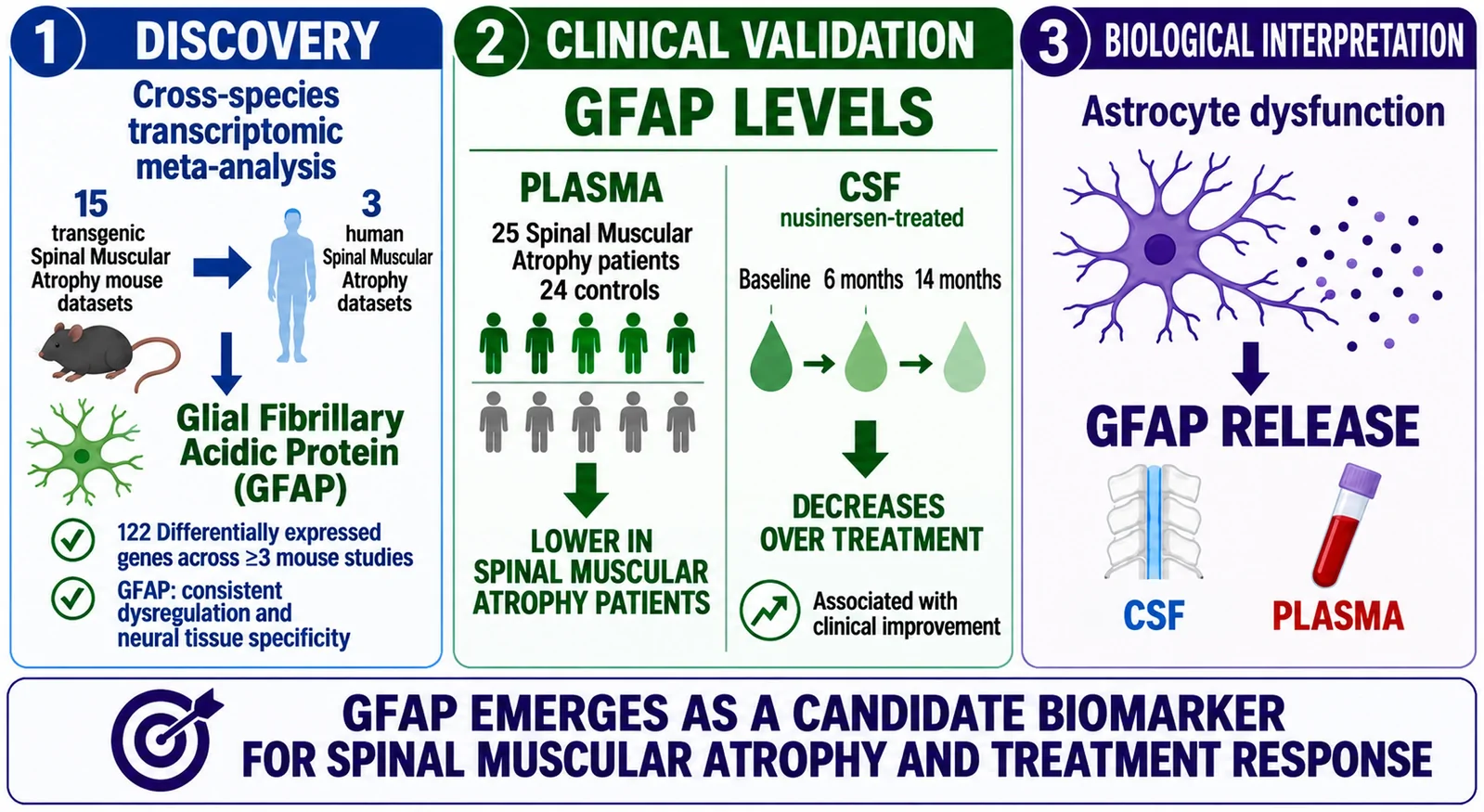

## Introduction

Spinal muscular atrophy (SMA) is a genetic neuromuscular disorder characterized by progressive muscle weakness due to the degeneration of motor neurons in the spinal cord. This is one of the most common autosomal recessive disorders, with an incidence of ∼1 in 10 000 live births and a carrier frequency of ∼1 in 40–60 individuals.^1-3^

SMA is caused by bi-allelic pathogenic variants in the survival motor neuron 1 (*SMN1*) gene, located on chromosome 5q13. *SMN1* encodes the SMN protein, which is essential for the survival of motor neurons. The severity of the disease is modulated by the number of copies of the *SMN2* gene, a paralog of *SMN1* that primarily produces low levels of functional SMN protein.^1,3^

SMA is classified into five types based on the age of onset and severity: type 0 (prenatal onset, most severe), type 1 (infantile, Werdnig–Hoffmann disease), type 2 (intermediate, Dubowitz disease), type 3 (mild, Kugelberg–Welander disease), and type 4 (adult-onset, mildest form). The disease spectrum ranges from severe infantile muscle weakness to mild adult-onset, with progressive loss of motor function in most cases.^1,2,4^

The treatment landscape for SMA has evolved significantly over the past decade. Historically, management was palliative, focusing on respiratory support, nutrition and physical therapy. However, the development of disease-modifying therapies (DMTs) has revolutionized care. Currently, three major treatments are available: (1) Nusinersen (Spinraza), an antisense oligonucleotide that enhances *SMN2* exon 7 inclusion to increase SMN protein production; (2) Onasemnogene abeparvovec (Zolgensma), a gene therapy that delivers a functional *SMN*1 gene via an adeno-associated virus (AAV9); and (3) Risdiplam (Evrysdi), an oral small-molecule splicing modifier that increases SMN protein levels. These treatments significantly improve motor function and survival but are costly, with Zolgensma being one of the most expensive drugs worldwide (∼$2.1 million per dose). Despite their efficacy, not all patients respond equally, and long-term outcomes remain under investigation.^5-8^

With the introduction of early treatment and newborn screening programmes, new phenotypes of SMA are emerging. Patients receiving early intervention exhibit milder symptoms or even near-normal development, complicating disease classification and prognosis. The emergence of new phenotypes and the variability in treatment response highlight the need for ongoing monitoring and adaptation of therapeutic strategies.^9^

To track disease progression and treatment response, biomarkers have become essential. The plasma levels of Neurofilament heavy chain (pNF-H) and Neurofilament light chain (NfL) have shown promise as indicators of axonal damage being elevated in untreated infantile SMA patients and changing with disease-modifying therapies.^10-12^ The lack of a major role of Neurofilament levels in later-onset SMA and its lack of coherence with the clinical response of disease-modifying drugs, with its levels being reduced by treatment with nusinersen^10^ and increased by treatment with onasemnogene abeparvovec^11^ limits its use in clinical practice. Despite these advances, the search for reliable biomarkers continues. Current studies focus on identifying novel molecular markers that correlate with disease severity, therapeutic response and long-term outcomes. The integration of biomarker research into clinical practice could refine treatment strategies, optimize patient monitoring and improve individualized care for SMA patients.^12^

This study aimed to investigate potential biomarkers for SMA severity and monitoring in an unbiased manner. Through a meta-analysis of transcriptomes of transgenic mice models of SMA, as well as from humans with SMA in a bioinformatic study, we identified glial fibrillary acidic protein (GFAP), a marker associated with astrocyte reactivation and neuroinflammation that was recently recognized as relevant in neurodegenerative processes, as one of the main hits. Subsequently, we assessed the NfL serum levels and GFAP serum and CSF levels in a clinical study to investigate their potential as biomarkers for disease severity, monitoring and treatment response to novel therapies.

## Methodology

### Ethics approval and consent to participate

The study was conducted in accordance with ethical guidelines and was approved by the institutional research ethics committee (GPPG-HCPA-2023-0054 CAAE 67592223.0.0000.5327). Informed consent was obtained from all participants or legal representatives, adhering to the ethical principles outlined in the Declaration of Helsinki.

### Study design

The study consisted of two phases: the first phase involved an unbiased bioinformatic study that conducted a meta-count of differential gene expression using datasets from humans and transgenic SMA mice models’ neural tissues; the second included a case–control study, followed by a cohort study of the SMA patients from a single centre, in which serum levels of the candidate biomarker (GFAP) and NfL as ‘positive control biomarker’ were analysed using single molecule array (SIMOA). Additionally, CSF levels of GFAP of the patients were evaluated immediately before (baseline) and up to 6 and 14 months after treatment with Nusinersen.

### Bioinformatic study

To unbiasedly identify potential SMA biomarkers, transcriptome datasets evaluating SMA disease were searched in publicly available repositories, including the Gene Expression Omnibus (GEO)^13^ and ArrayExpress (AE).^14^ The following keywords were used: ‘(SMA) OR (Spinal muscular atrophy) OR (SMN1) OR (SMN2)’. No restrictions were applied regarding tissue or cell type, allowing for the inclusion of datasets from studies involving patients, cellular models, or transgenic models of the disease analysed using microarray or RNA-Seq methodologies. Studies with fewer than three samples per group, without control groups or without raw data available in the databases were excluded. Two independent reviewers (K.L.S.O. and A.C.W.) conducted the database search, with a third reviewer (J.A.M.S.) resolving disagreements regarding dataset inclusion or exclusion. Due to study heterogeneity, a meta-count analysis was performed, and genes differentially expressed compared to controls in at least three studies were selected for further investigation.

### Clinical study

The first phase of the clinical study was a cross-sectional case–control study in which serum levels of GFAP and NfL were analysed using SIMOA. Additionally, in the case group, clinician-rated outcomes (ClinRO) were assessed, including the Children’s Hospital of Philadelphia Infant Test of Neuromuscular Disorders (CHOP-INTEND), Hammersmith Infant Neurological Examination (HINE), Hammersmith Functional Motor Scale–Expanded (HFMSE), and Revised Upper Limb Module (RULM). SMA functional status was categorized as non-sitters; sitters (defined as able to sit without support, stand alone or stand with assistance); and walkers (defined as able to walk independently). In the second phase, a longitudinal cohort study was conducted exclusively with SMA patients. This cohort involved a 12-month follow-up period during which biomarker analysis was repeated. Additionally, GFAP levels in the cerebrospinal fluid (CSF) were analysed in 12 of the 25 included patients (nine with SMA type 1 and three with types 2 or 3) and two additional pre-symptomatic individuals (one with 2 and the other with 4 *SMN2* copies) immediately before and up to 14 months after treatment with nusinersen. Participants were consecutively recruited from May to October 2021 at the Neuromuscular Genetics outpatient clinic of Hospital de Clínicas de Porto Alegre (HCPA). The study included individuals with a confirmed genetic diagnosis of SMA types 1, 2 or 3 undergoing regular follow-up. The control group consisted of healthy participants matched to the case group by sex and age. Detailed eligibility criteria were previously described.^15^

### Biomarkers analysis

Three millilitre of serum samples were obtained from peripheral venous blood of participants and controls and placed in serum separator tubes. The samples were left at room temperature (18°C–25°C) for 30–45 min to clot, then centrifuged at 1000 × *g* for 10 min at a temperature of 23°C. Care was taken to avoid haemolysis and the separated serum was stored in a freezer at −80°C until analysis. Serum levels of GFAP and NfL were measured according to the standard protocol using the Simoa® Neurology 2-Plex B Kit (103520), while CSF GFAP levels were measured by ELISA (Invitrogen, EEL079).

### Statistical analysis

#### Datasets processing and meta-analysis

The microarray studies were manually extracted through GEO/AE and later analysed with the affy package^16^ in R v4.2.2 (ref. ^17^) and RStudio v2022.7.2.576 software applying a robust multiarray average normalization. The raw data from the RNA-Seq datasets were processed on the useGalaxy server^18^ as follows: .fastq quality was evaluated in the FastQC,^19^ alignment was performed using the Bowtie2^20^ and quantification using the featurecounts^21^ tools. DGE analysis was conducted for each dataset in R and RStudio using the limma package^22^ for microarray studies and edgeR^23^ package for the RNA-Seq assays. The scripts used in the analyses are available at https://github.com/bioinfo-hcpa/codes_and_scripts/tree/main/transcriptomics. Principal component analysis (PCA) was performed for each dataset to evaluate the heterogeneity of the samples. Samples’ normality was assessed by performing the Shapiro-Wilk Test in R; if the *P*-value < 0.05, non-parametric tests were performed. The surrogate variable analysis normalization was performed to reduce batch effects. *P*-values were corrected by the false discovery rate method. The resulting genes from the DGE analysis with logFC ≥ 1 and corrected *P*-value ≤ 0.05 were considered upregulated, whilst genes with logFC ≤ −1 and corrected *P*-value ≤ 0.05 were considered downregulated. Meta-analysis was performed using the metavolcano package in R.^24^ To integrate human and mouse data, orthologs were assessed using the BioMart^25^ R package by submitting the *Mus musculus* gene (GRCm9) and searching the *Homo sapiens* ortholog (GRCh38.p14). Only orthologs with high confidence were considered, provided by Biomart according to the Ensembl orthology quality control.

#### Clinical study analysis

Statistical tests were selected according to the distribution of data given by Shapiro–Wilk test and histograms. Most variables in the study did not follow a normal distribution and are presented as median (interquartile ranges), except for CHOP-INTEND, HFMSE, and RULM, which are reported as mean (95% confidence interval, CI). Comparisons between paired cases and controls were performed with Wilcoxon signed-rank test or chi-square. Comparisons among SMA types, functional status and *SMN2* copy numbers were performed with Kruskal–Wallis test. Comparisons between SMA-treated and untreated subjects at baseline were performed with Mann–Whitney U-test. Changes in GFAP and NfL serum levels from baseline to 12 months of follow-up were performed with Wilcoxon signed-rank test. Correlations between GFAP and NfL levels at baseline with age, age at onset, reported disease duration and the clinical outcome assessment (COAs) were performed with Spearman correlation test. Additionally, we performed a Spearman correlation test to evaluate the association between biomarker level changes from baseline to 12 months and time since onset of treatment (TSOT). TSOT was defined as the number of days of treatment at the time of baseline sample collection with positive values indicating that treatment had already started before baseline, zero meaning that treatment began at the baseline visit and negative values that treatment started after baseline. Untreated patients were censored for this last analysis. For comparison of CSF GFAP levels at baseline, 6 months and 14 months after Nusinersen treatment, Generalized Estimating Equations model was used with log_10_-transformed data and a gamma distribution. Statistical analyses and graphical visualizations were performed using IBM SPSS Statistics version 20, except for violin plots with embedded boxplots and individual data points, which were generated using the ggplot2 package in R version 4.5.1.

## Results

### Bioinformatic study

The initial search identified 152 studies, of which 17 were analysed, including samples of neural, muscular and whole blood origin. Two PCAs were conducted: one for seven RNA-Seq studies in transgenic mice and another for three RNA-Seq studies in humans with SMA (Supplementary Figs 1 and 2).

These analyses showed a clear separation between neural, muscular and blood sample components, making it inadequate to analyse data from different tissues together. The dataset GSE51735 in mice exhibited markedly different behaviour in the PCA and was excluded from the analysis. Microarray datasets were later added to this phase.

Therefore, the present study focused on the transcriptome datasets from neural samples. Initially, we performed a meta-analysis of 15 datasets (Supplementary Appendix) from transgenic mouse studies (some represented multiple times due to different neuronal populations) and three datasets from SMA patient samples. We then analysed differentially expressed genes in transgenic mouse models considering: (i) the total dataset; (ii) only RNA-Seq data; a dataset redundancy reduction approach where only one nervous system region (lumbar spinal cord) was considered, using either (iii) RNA-Seq or microarray or (iv) only RNA-Seq data. The Venn diagram in Supplementary Fig. 3 highlights the heterogeneity of these results, with significant variations in gene lists depending on the analysis method. Given this variability, we opted for a non-parametric approach using the meta-count method, which evaluates the recurrence of differentially expressed genes across studies. The meta-count analysis of the 15 transgenic mouse datasets identified 134 differentially expressed genes in at least 3 of the 15 studies, of which 122 were orthologous in humans (Supplementary Table 1). The pathway enrichment analysis according to the gene ontology is presented in Supplementary Fig. 4. Among these 122 genes, 24 showed altered expression in at least one human SMA datasets (Supplementary Table 2), and only five exhibited consistent expression changes across mice studies (either all upregulated or all downregulated). Two of these genes were specifically expressed in the central nervous system according to the Genotype-Tissue Expression (GTEx) Project^26^ and The Human Protein Atlas,^27^ including *GFAP*.

Given the identification of *GFAP* as a hit, we reanalysed the expression levels of genes related to astrocytic function in the transgenic mouse datasets, including *S100b, Glul, Aqp4, Glast (Slc1a3), Glt-1 (Slc1a2), Aldh1l1 and Ykl-40 (Chi3l1). Aldh1l1, S100b and Slc1a2* were differentially expressed in only one study, whereas *Ykl-40 (Chi3l1), Aqp4* and Slc1a3 were differentially expressed in two of the 15 GEO datasets. *kl-40 (Chi3l1)* showed consistent upregulation, *Aqp4* showed consistent downregulation, while *Slc1a3* exhibited upregulation in one dataset and downregulation in another. Detailed results are provided in Supplementary Table 3. Of note, pathway enrichment analysis (Supplementary Fig. 4) depicted gliogenesis as one of the relevant pathways of SMA.

### Clinical study

A total of 49 participants were included in the study, comprising 25 homozygous cases with the common *SMN1* deletion and 24 healthy controls. The detailed clinical, genetic and demographic characteristics of the participants are presented in Table 1. At baseline, 10 out of 25 (40%) SMA cases were receiving SMN-targeted DMTs. All of them received Nusinersen and 3 of 10 (12% of the whole cohort) transitioned to Onasemnogene abeparvovec. During the 12-month follow-up period, four additional subjects initiated treatment, and one patient switched from Nusinersen to Onasemnogene abeparvovec.

**Table 1:** Demographics, clinical and genetic characteristics of the study sample at baseline

Variable	Controls (*n* = 24)	SMA subjects (*n* = 25)
Male	12 (50%)	12 (48%)
Age, mo	126.5 (211)	132 (227)
Age at onset, mo	-	8 (11)
Disease duration, mo	-	126 (220)
SMA type		Type 1—11/25 (44%)
	-	Type 2—9/25 (36%)
		Type 3—5/25 (20%)
*SMN2* copy number		2 copies—12/25 (48%)
	-	3 copies—10/25 (40%)
		4 copies—3/25 (12%)
Walkers	24/24 (100%)	2/25 (8%)
CHOP-INTEND (*N* = 16)^a^	-	24.3 (14.8–33.8)
HINE (*N* = 15)	-	2 (5)
HFMSE (*N* = 8)^a^	-	19.5 (8.5–30.4)
RULM (*N* = 6)^a^	-	22.8 (12.1–33.5)
On DMTs	-	10 (40%)^b^
Use of Nusinersen	-	10 (40%)
Use of Onasemnogene abeparvovec	-	3 (12%)^c^

Data are shown as median (interquartile range) or frequencies (percentages), except for BMI, which is shown as mean (95% confidence interval).

CHOP-INTEND, Children’s Hospital of Philadelphia Infant Test of Neuromuscular Disorders; DMT, disease-modifying therapies; HINE, Hammersmith Infant Neurological Examination; HFMSE, Hammersmith Functional Motor Scale–Expanded; Mo, months; RULM, Revised Upper Limb Module; SMA, spinal muscular atrophy.

^a^Data are shown as mean (95% confidence interval).

^b^Four additional patients started DMTs at baseline or between baseline and the follow-up evaluation.

^c^One patient transitioned from Nusinersen to Onasemnogene Abeparvovec in between baseline and 12 mo of serum collection.

### Serum levels of biomarkers

#### Glial fibrillary acidic protein

**Figure 1:**
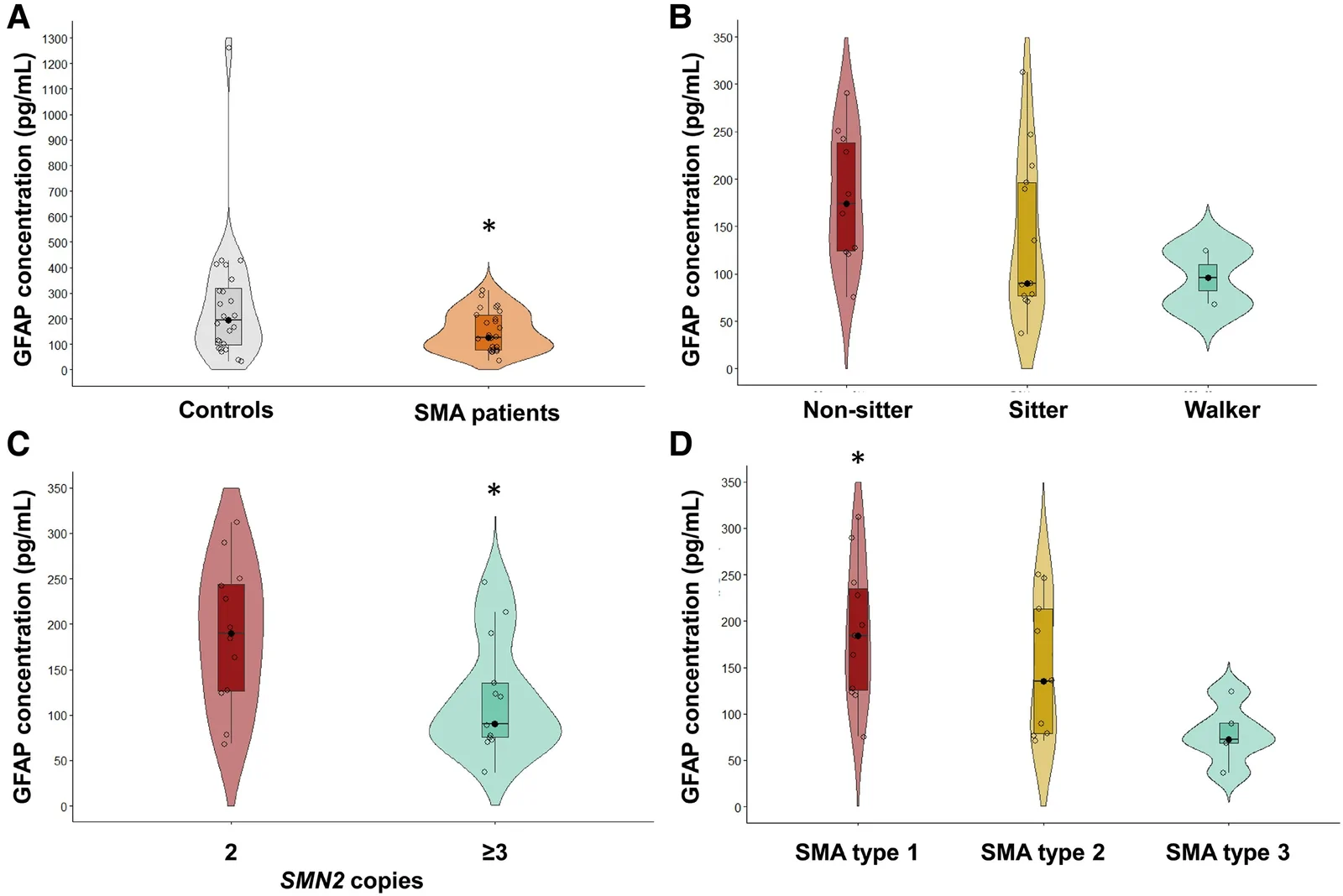
**GFAP serum levels at baseline**. (**A**) Paired comparisons between cases and controls were performed using the Wilcoxon signed-rank test (*W* = 64, *P* = 0.014). *N* = 25 for SMA and 24 for controls. (**B**) Comparisons among functional statuses were performed using the Kruskal–Wallis test (*H*(2) = 3.14, *P* = 0.208), *n* = 10 for non-sitter, 13 for sitter and 2 for walker. (**C**) Comparisons between *SMN2* copy numbers were performed using the Mann–Whitney U-test (*U* = 129.0, *P* = 0.03), *N* = 12 for 2 copies and 13 for 3 or more copies. (**D**) Comparisons among SMA types were performed using the Kruskal–Wallis test, *post hoc* comparisons were performed with Dunn’s test with Bonferroni correction comparisons (*H*(2) = 7.119, *P* = 0.028). In the *post hoc* analysis, the difference was observed between type 1 and type 3 (*Z* = 10.582, adjusted *P* = 0.023). *N* = 11 for type 1, 9 for type 2, and 5 for type 3. Violin plots with embedded box plots are shown, and each dot represents an independent patient sample. *P < 0.05.

Serum GFAP levels were reduced in SMA patients (median: 127.5 pg/ml; interquartile range: 142.8) compared to controls (median: 194.1 pg/ml; interquartile range: 253.6; *P* = 0.014, Fig. 1A). Differences were observed across SMA types, with higher levels in type 1 patients (*P* = 0.028, Fig. 1D) and in those with two *SMN2* copies compared to those with ≥3 copies (*P* = 0.03, Fig. 1C). No significant differences were found based on functional status (*P* = 0.208; Fig. 1B). Treated patients had higher GFAP levels (median: 201.3 pg/ml; interquartile range: 88.3) compared to treatment-naive patients (median: 89.7 pg/ml; interquartile range: 63.0; *P* = 0.01, Supplemental Fig. 5) at baseline.

**Figure 2:**
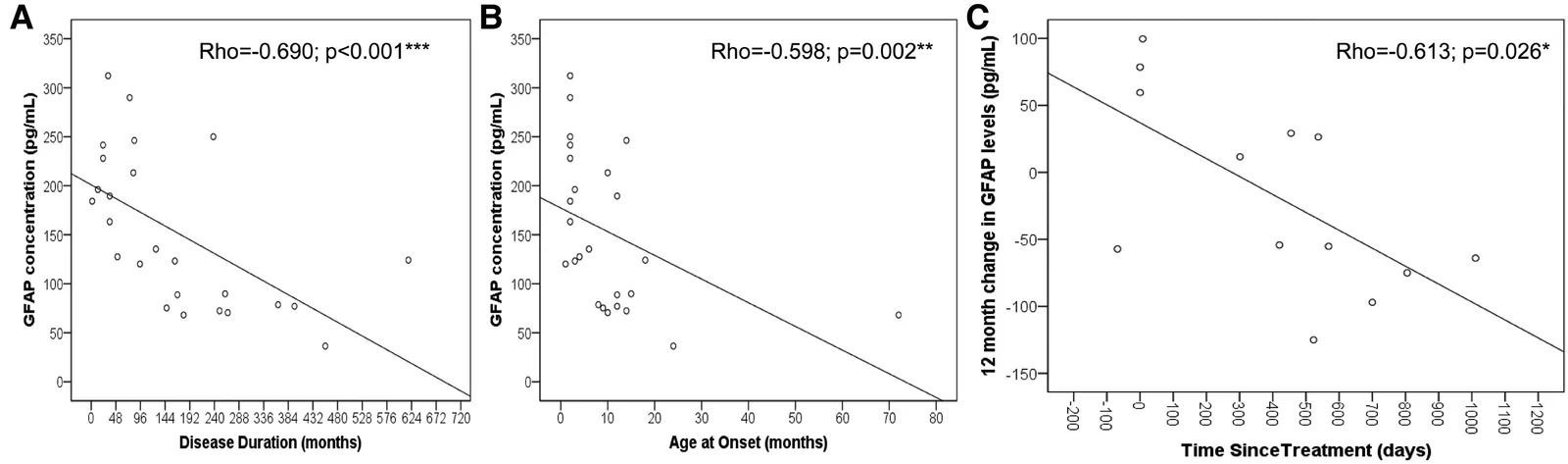
**GFAP serum levels correlation with clinical severity outcomes and time since treatment.** (**A**) Correlation of GFAP serum levels with disease duration (months), *N* = 25. (**B**) Correlation of GFAP serum levels with age at onset (months), *N* = 25. (**C**) Correlation between the difference in GFAP serum levels (baseline versus 12 months) and the time since treatment onset (days), *N* = 13. Correlations were performed using the Spearman rank test. Dots represent individual patient data points. *P < 0.05. **P < 0.01. ***P < 0.001.

GFAP levels correlated with disease duration (Rho = −0.690, *P* < 0.001, Fig. 2A) and age at onset (Rho = −0.598, *P* = 0.002, Fig. 2B). No significant correlations were found between GFAP levels and COAs (Supplementary Table 4). GFAP levels did not change significantly after 12 months (*P* = 0.627), but the variation in levels correlated with treatment duration, showing an inverse relationship between GFAP increase and TSOT (Fig. 2C).

#### Neurofilament light chain

**Figure 3:**
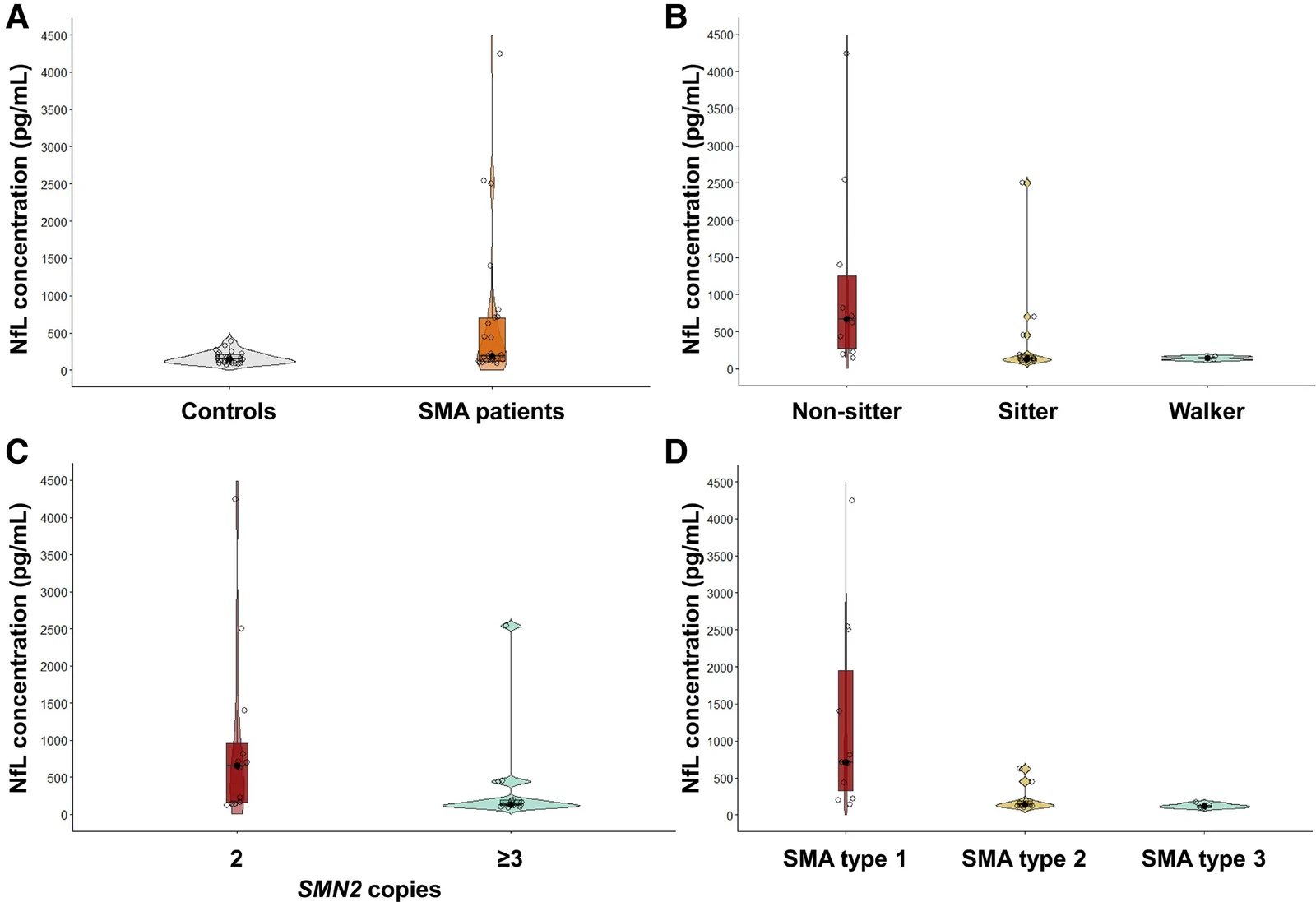
**NfL serum levels at baseline.** (**A**) Paired comparisons between cases and controls were performed using the Wilcoxon signed-rank test (*W* = 241, *P* = 0.009). *N* = 25 for SMA and 24 for controls. (**B**) Comparisons among functional statuses were performed using the Kruskal–Wallis test (*H*(2) = 9.326, *P* = 0.009), *n* = 10 for non-sitter, 13 for sitter, and 2 for walker. In the *post hoc* analysis performed using Dunn’s test with Bonferroni correction, a difference was observed only between non-sitters and sitters (*Z* = 9.077, adjusted *P* = 0.010). (**C**) Comparisons between SMN2 copy numbers were performed using the Mann–Whitney U-test (*U* = 34.5, *P* = 0.016), *N* = 12 for 2 copies and 13 for 3 or more copies. (**D**) Comparisons among SMA types were performed using the Kruskal–Wallis test (*H*(2) = 14.02, *P* = 0.001). In the *post hoc* analysis performed using Dunn’s test with Bonferroni correction, differences were observed between type 1 and type 2 (*Z* = 9.278, adjusted *P* = 0.015) and between type 1 and type 3 (*Z* = 13.3, adjusted *P* = 0.002). *N* = 11 for type 1, 9 for type 2, and 5 for type 3. Violin plots with embedded box plots are shown, and each dot represents an independent patient sample. *P < 0.05; **P < 0.01; ***P < 0.001.

Serum NfL levels were elevated in SMA patients (median: 187.0 pg/ml; interquartile range: 581.0) compared to controls (median: 150.0 pg/ml; interquartile range: 123.0; *P* = 0.009, Fig. 3A). Differences were observed based on functional status, with higher levels in non-sitters (*P* = 0.009, Fig. 3B), SMA types (higher in type 1, *P* = 0.001, Fig. 3D) and *SMN2* copy number (higher in patients with two *SMN2* copies compared to those with ≥3 copies, *P* = 0.016, Fig. 3C). No significant differences were found between treated (median: 204.5 pg/ml; interquartile range: 758.0) and treatment-naive patients (median: 167.9 pg/ml; interquartile range: 499.0; *P* = 0.683) at baseline.

**Figure 4:**
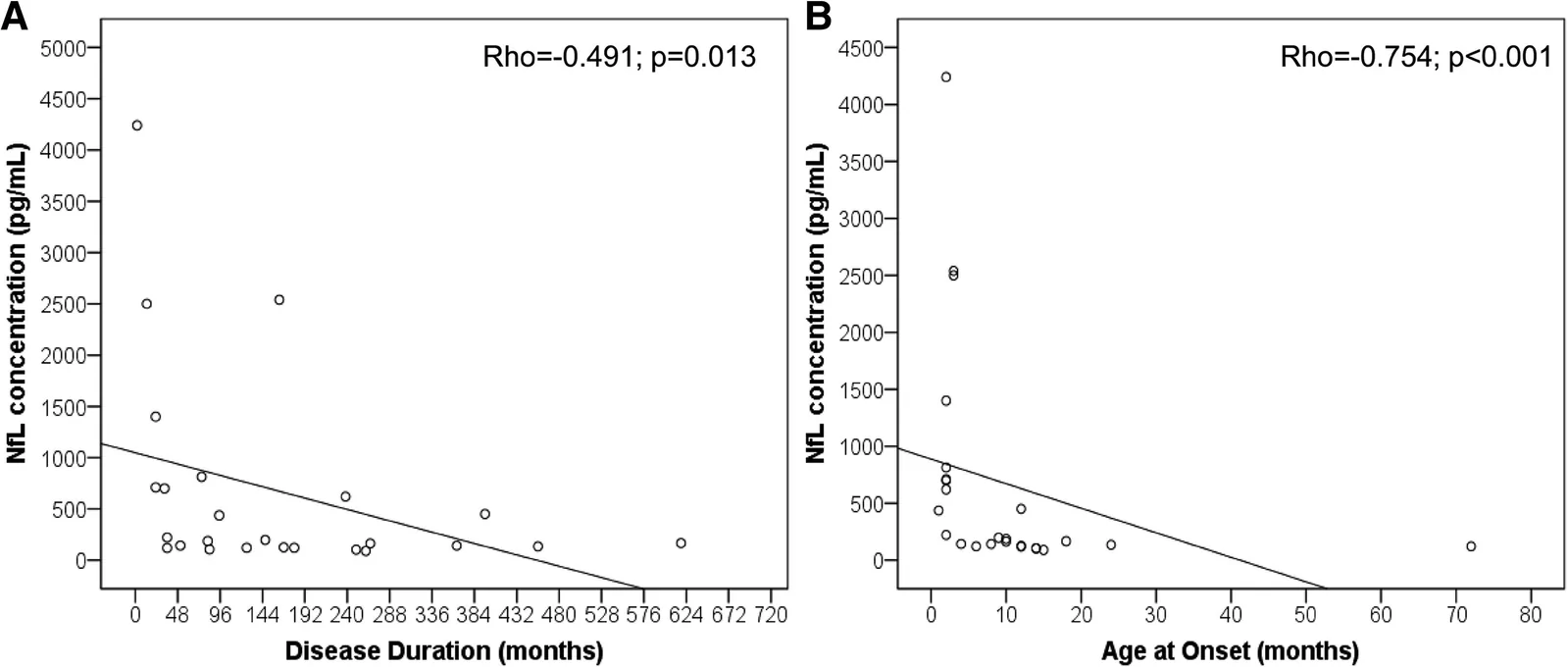
**NfL serum levels correlation with clinical severity outcomes and time since treatment.** (**A**) Correlation of NfL serum levels with disease duration (months), *N* = 25 (**B**) Correlation of NfL serum levels with age at onset (months). Correlations were performed using the Spearman rank test, *N* = 25. Dots represent individual patient data points.

NfL levels correlated with disease duration (Rho = −0.491, *P* = 0.013, Fig. 4A) and age at onset (Rho = −0.754, *P* < 0.001, Fig. 4B). No significant correlations were found between NfL levels and COA severity scores (Supplementary Table 2).

NfL levels changed over 12 months (*P* = 0.023), increasing from baseline (median: 164.0 pg/ml; interquartile range: 177.5) to follow-up (median: 180.0 pg/ml; interquartile range: 229.0; *P* = 0.023). However, there was no correlation between NfL variation and TSOT (Rho = 0.110, *P* = 0.720).

#### GFAP and NfL

GFAP and NfL serum levels showed a direct correlation (Rho = 0.375, *P* = 0.013) at baseline.

#### CSF GFAP levels after Nusinersen therapy

GFAP levels in the CSF were analysed in 12 out of 25 cases in the study (median age = 43 months, IQR = 79), including nine patients with SMA type 1 and three patients with type 2, as well as in two additional pre-symptomatic individuals—one with 2 copies of *SMN2* and the other with 4 copies of *SMN2*. No significant differences in CSF GFAP levels were observed at baseline compared to 6 months (*B* = 0.02, 95% CI −0.067 to 0.108, *P* = 0.650) or 14 months (*B* = −0.086, 95% CI −0.215 to 0.044, *P* = 0.195, Supplementary Fig. 6A) of treatment.

However, when analysing only patients with SMA type 1 or pre-symptomatic individuals predicted to develop SMA type 1, a reduction in GFAP levels was observed at baseline compared to 14 months of treatment (*B* = −0.153, 95% CI −0.303 to −0.004, *P* = 0.045), while no significant difference was found after 6 months of therapy (*B* = 0.052, 95% CI −0.033 to 0.138, *P* = 0.045, Supplementary Fig. 6B). Supplementary Fig. 7A depicts the longitudinal evolution of CSF GFAP levels and HINE scores at the individual patient level, while Supplementary Fig. 7B presents the corresponding trajectories of CSF GFAP levels and CHOP-INTEND scores, assessed at baseline, 6 months, and 14 months. Together, these figures illustrate potential inverse trends between GFAP concentrations and clinical improvement across patients.

## Discussion

In this study, an integrated and unbiased bioinformatics approach was employed to identify candidate biomarkers for SMA through gene expression analysis of transcriptomic datasets from neural samples of transgenic mouse models and SMA patients. This strategy highlighted GFAP as a potential biomarker for SMA. Clinical validation identified lower serum GFAP levels, which were associated with disease type, *SMN2* copy number, and treatment status. In SMA type 1, CSF GFAP levels decreased over time with DMT. Serum NfL levels were assessed as a validation tool, given that NfL has been extensively demonstrated and validated as a biomarker for infantile SMA in recent years.^10^ The results suggest a complementary relationship between these biomarkers, revealing a mutual correlation that implicates both neurons and astrocytes as key players in the neurodegenerative process of SMA.

GFAP is an intermediate filament protein specific to astrocytes. In response to acute CNS trauma, ischaemia, or neurodegenerative diseases, astrocytes adopt a reactive phenotype characterized by upregulation and reorganization of GFAP.^28^ This protein has emerged as a promising biomarker in various neurological disorders, particularly Alzheimer’s disease, where it is considered a non-invasive marker of early reactive astrogliosis.^29-32^ Furthermore, pathogenic variants in the *GFAP* gene are linked to Alexander disease, a leukodystrophy characterized by astrocytic dysfunction and GFAP aggregates.^33,34^

From a mechanistic perspective, astrocytes are increasingly recognized as active contributors to neurodegeneration through non-cell-autonomous mechanisms.^35,36^ Our transcriptomic analysis revealed upregulation of GFAP across transgenic SMA datasets and downregulation in a SMA patient iPSC-derived spinal motor neurons dataset, a more complex scenario than the reactive astrogliosis typically observed in amyotrophic lateral sclerosis or Alzheimer’s disease patients, which is usually accompanied by increased GFAP levels.^37-39^ This finding may suggest an attenuated or dysfunctional glial response in SMA, potentially due to intrinsic SMN-related deficits in astrocytic maturation and function, as previously demonstrated.^35,40^ Notably, these studies showed that SMA astrocytes exhibit defects in calcium homeostasis and that motoneuron-astrocyte co-cultures present significant reductions in synapse formation and synaptic transmission.^36^ Furthermore, viral-mediated restoration of SMN specifically in astrocytes improved survival in severe and intermediate SMA mouse models.^35^

There is a scarcity of studies evaluating glial markers in SMA patients.^12^ Olsson *et al*. assessed neurochemical biomarkers in the CSF of 12 children with SMA type 1 before and after nusinersen treatment in a single-centre study.^41^ The primary focus was on NfL, although tau and GFAP were also measured. At baseline, GFAP and NfL were elevated. After eight doses of nusinersen, both decreased; however, only NfL reduction correlated with ClinROs improvements. These findings suggest GFAP may be less dynamic and could reflect more chronic astrocytic involvement. Importantly, this study included a control group for CSF analysis, whereas our study did not. Of note, the CSF GFAP levels in our cohort (Supplementary Fig. 6) were higher than those reported in Olsson *et al*., where the mean was 236 ± 44 pg/ml. Nonetheless, both studies demonstrated reduced CSF GFAP levels following nusinersen in SMA type 1.

A multicentre German study analysed CSF GFAP in 30 healthy controls and 79 SMA patients (58 adults and 21 children), of whom only seven had SMA type 1.^42^ No significant differences in CSF GFAP were found overall, though after adjusting for age, higher GFAP was associated with greater disease severity. Over 14 months of nusinersen treatment, CSF GFAP remained unchanged, while NfL levels significantly decreased. The authors concluded that while CSF GFAP may not be a robust SMA biomarker, elevated levels could reflect glial reactivity. Notably, our cohort included 44% SMA type 1 patients, compared to only 8% in the German study. This difference in sample composition, with our cohort enriched for more severe, rapidly progressing cases, likely accounts for discrepancies in findings. It is worth noting that even NfL levels in the German cohort did not differ significantly from controls.^42^ Both studies, however, observed higher GFAP levels in more severely affected patients, supporting its potential as a marker of disease severity. Another key distinction lies in the biofluid analysed: the German study assessed CSF GFAP, while we evaluated plasma GFAP. In Alzheimer’s disease, plasma GFAP has shown greater sensitivity to early pathological changes than CSF GFAP,^31^ suggesting plasma GFAP may be a more dynamic and accessible biomarker. Therefore, differences in biofluid may also contribute to discrepancies in GFAP findings across studies.

Higher GFAP levels in SMA type 1 patients with two *SMN2* copies may indicate more pronounced astrocytic activation in severe cases, which is in line with GFAP upregulation found in datasets from transgenic mouse models with severe phenotype. The reduction in CSF GFAP with DMTs in this subgroup^41^ supports this idea and suggests diminished astrocytic reactivity with treatment. This is further corroborated by the inverse correlation between serum GFAP changes and time since treatment initiation, indicating greater GFAP reduction in patients on longer-term DMTs. It is also noteworthy that alterations in at least one dataset were observed for six other astrocytic markers, and that enrichment of the gliogenesis pathway was identified in SMA, further reinforcing the role of glia in the condition and suggesting that future studies should perform a broader analysis of additional glial markers, now guided by hypothesis-driven approaches.

The similarity in trends between GFAP and NfL, including higher levels in more severe phenotypes and reductions with DMTs, along with their correlation in this study, suggests GFAP may also serve as a marker of neurodegeneration rate. This supports the view that SMA pathophysiology involves both neuronal and astrocytic dysfunction, in line with modern perspectives on neurodegenerative disease mechanisms.^43^ Furthermore, the observation that CSF GFAP only changed after 14 months of treatment implies a slower glial response to DMTs.

In our cohort, serum NfL levels were elevated in SMA patients, especially in those with more severe phenotypes such as type 1 and non-sitters, consistent with NfL’s role as a marker of axonal damage and previous findings linking higher NfL levels to SMA severity.^10,44-46^ The significant correlation between GFAP and NfL reinforces the concept of concurrent astrocytic and neuronal involvement, enhancing the credibility of GFAP as a complementary biomarker.

The recognition that glial cells play a central role in the degenerative process of the disease,^43,47^ and that the absence of SMN protein directly impairs their function, may be crucial for the development of future gene delivery therapies for SMA. Notably, there are significant differences in the ability of viral vectors and promoters to penetrate glial cells and neurons in such therapies.^48^

In summary, although speculative, we suggest that GFAP levels may be reduced in SMA due to astrocytic loss and dysfunction driven by SMN deficiency, while higher levels in SMA type 1 may reflect reactive changes in remaining astrocytes due to rapid neurodegeneration. The observed GFAP reduction with DMT use further supports this hypothesis.

### Study limitations

One of the limitations of our study is that it is a single-centre study with a relatively small sample size. At the time the study was designed, there were no prior data on serum GFAP in SMA to allow for a sample size calculation. We conducted an exploratory study, considering the differences between cases and controls in serum GFAP as the main outcome, without statistical corrections for multiple comparisons. Despite the limitations of our convenience sample, we identified significant differences in GFAP levels, and these results were supported by the meta-count analysis of DGE studies in datasets from transgenic mice models and human samples. The sample size for CSF analysis was small, and untreated CSF controls were not available due to the paediatric population, making it not feasible to obtain an adequate control group with CSF samples for comparison. It is also important to note that quantification methods differed between serum (SIMOA) and CSF (ELISA), introducing potential analytical variability. However, existing studies show a strong correlation between serum and CSF GFAP, which helps mitigate this issue. The heterogeneity of the SMA population, encompassing different SMA subtypes, and the recruitment during the COVID-19 pandemic resulted in a limited number of patients with complete ClinROs available. Future studies with larger sample sizes will be crucial to assess the correlations between GFAP levels and ClinROs more comprehensively across SMA types. Also, the difference in GFAP levels between treated and untreated patients at baseline, as well as the inverse correlations with disease duration and age of onset, likely reflect the influence of SMA type on GFAP levels. Most of the treated patients in the study had SMA type 1, and those with shorter disease duration and earlier symptom onset were also those with type 1 SMA (who exhibit higher GFAP levels). The study did not have sufficient power to perform statistical corrections for this purpose, underscoring the importance of future studies more thoroughly evaluating the effect of DMTs on the serum biomarker by comparing results of treatment-naive patients before and after treatment, as well as further exploring the association with disease duration or even age of symptom onset, in separate comparisons for each subtype of the condition.

## Conclusion

In conclusion, our findings support GFAP and NfL as promising, complementary biomarkers for SMA. Their distinct expression patterns reflect different aspects of SMA pathophysiology, axonal and glial involvement, and their correlation provides a more integrated understanding of disease activity. The combined use of GFAP and NfL may enhance biomarker sensitivity, particularly in severe cases, and prove valuable for long-term monitoring. These results highlight the need for further investigation in larger cohorts with harmonized methodologies. Additionally, they suggest the future development of multimodal biomarker panels to optimize SMA care and therapeutic decision-making.

## Supplementary Material

fcag350_Supplementary_Data

## Data Availability

The datasets used in this study are all publicly available in GEO and ArrayExpress, with the corresponding accession codes provided in Supplementary Appendix. Data not provided in the article because of space limitations may be shared (anonymized) at the request of any qualified investigator for purposes of replicating procedures and results.
