# Supplementary material for: Unbiased bioinformatic and clinical analysis identifies glial fibrillary acidic protein as a potential biomarker in spinal muscular atrophy
Source: Brain Commun. 2026 Sep 11;8(5):fcag350. doi: 10.1093/braincomms/fcag350 (PMC13613212; doi:10.1093/braincomms/fcag350)
Supplement: Supplementary file 1 [file fcag350_supplementary_data.zip › Supplementary_figure_legends_ and_Appendix.docx]

**Appendix**

The initial meta-analysis included 15 datasets from transgenic mice: GSE102204_AllSamples (RNA-Seq, datasets early and early-symptomatic analyzed together), GSE112771 (RNA-Seq), GSE115706_Facial (RNA-Seq, Facial dataset), GSE115706_Hypoglossal (RNA-Seq, Hypoglossal dataset), GSE115706_Lumbar (RNA-Seq, Lumbar dataset), GSE115706_Oculomotor (RNA-Seq, Oculomotor dataset), GSE115706_Red (RNA-Seq, Red dataset), GSE115706_Vagus (RNA-Seq, Vagus dataset), GSE128129_AllSamples (RNA-Seq, datasets developmental stage P2, P6, and P10 analyzed together), GSE19674 (array), GSE56284 (RNA-Seq), GSE81245_BrainStem (array, Brain Stem dataset), GSE81245_DorsalRoot (array, Dorsal Root dataset), GSE81245_SpinalCord (array, Spinal Cord dataset), and GSE87281 (RNA-Seq). A total of 36 differentially expressed genes were identified (-1 ≥ logFC ≥ 1).

The meta-analysis of studies involving SMA patients included three datasets: GSE65470 (array), GSE108094 (RNA-Seq), and GSE159642_SpinalCord (RNA-Seq, Spinal Cord dataset). A total of 1.197 differentially expressed genes were identified (-1 ≥ logFC ≥ 1).

**Supplemental Figures Legends**

**Supplemental Figure 1:** **Principal Component Analysis of the seven studies in mice.** The analyzed datasets were GSE51735, GSE56284, GSE102204, GSE112771, GSE128129, GSE150510, and GSE158790, including 85 samples overall. Studies involving neuronal cell types are shown in blue, while muscular studies are shown in purple. Dark shades represent case samples (neuronal or muscular), whereas light shades represent controls. Subsequently incorporated into the final set of studies: GSE115706 and GSE87281 (RNA-Seq); GSE19674, GSE81245, and GSE150517 (arrays).

**Supplemental Figure 2:** **Principal Component Analysis of the three studies in SMA patients.** The analyzed datasets were GSE108094, GSE159642, and GSE174056, including 45 samples overall. Studies with neural samples are shown in blue, muscular samples in purple, and blood samples in red. Dark shades represent case samples, while light shades represent controls. Subsequently incorporated into the final set of studies: GSE65470 and GSE58316 (arrays).

**Supplemental Figure 3:** Venn diagram showing the number of differentially expressed genes across four data subsets: RNAseq only lumbar (blue), Only RNAseq (red), Only lumbar (green), and Original (yellow). (1) RNAseq only lumbar: only RNA-Seq and the Lumbar dataset from GSE115706; (2) Only RNAseq: only RNA-Seq and all datasets from GSE115706; (3) Only lumbar: RNA-Seq and array, only the Lumbar dataset from GSE115706; and (4) Original: all data. logFC ≥ 1 and corrected P-Value ≤ 0.05 were considered the thresholds for being considered a differentially expressed gene.

**Supplemental Figure 4:** **Major pathways enriched in transgenic SMA models**. Pathway enrichment analysis with the 122 human ortholog genes, differentially expressed in at least three of the 15 studies, according to Gene Ontology (GO).

**Supplemental Figure 5 – GFAP levels at baseline and treatment status.** Comparisons between SMA treated, and untreated subjects at baseline were performed with Mann–Whitney U-test, (U=121, p=0.01), N=15 for non-treated and 10 for treated patients at baseline. **p=0.01. Violin plots with embedded box plots and individuals are shown, and each dot represents an independent patient sample.

**Supplemental Figure 6: CSF levels of GFAP after nusinersen treatment.** (A) Log10 GFAP levels at baseline and after 6 and 14 months of nusinersen therapy (N of individuals=14). No significant differences in CSF GFAP levels were observed at baseline compared to 6 months (B=0.02, 95% CI -0.067 to 0.108, Wald Chi-Square=0.206, p=0.650) or 14 months (B=-0.086, 95% CI -0.215 to 0.044, Wald Chi-Square=1.676, p=0.195). Eight samples were evaluated at baseline, 9 after 6 months, and 10 after 14 months. (B) Log10 GFAP levels at baseline and after 6 and 14 months of nusinersen therapy in patients with SMA type 1 and pre-symptomatic individuals predicted to develop SMA type 1 (N of individuals=10). A reduction in GFAP levels was observed at baseline compared to 14 months of treatment (B=-0.153, 95% CI -0.303 to -0.004, Wald Chi-Square=4.031, p=0.045), while no significant difference was found after 6 months of therapy (B=0.052, 95% CI -0.033 to 0.138, Wald Chi-Square=1.437, p=0.231). Six samples were evaluated at baseline, 5 after 6 months, and 5 after 14 months. Generalized Estimating Equations models were used with Log10-transformed data and a gamma distribution. *p<0.05.

**Supplemental Figure 7**. **Longitudinal evolution of CSF GFAP levels, HINE and CHOP-INTEND scores in SMA patients treated with Nusinersen.** The spaghetti plot illustrates potential inverse trends between GFAP levels and clinical improvement across patients by displaying the individual trajectories of cerebrospinal fluid (CSF) glial fibrillary acidic protein (GFAP) levels (solid lines, left Y-axis, log10-transformed) and A) Hammersmith Infant Neurological Examination (HINE) scores (dashed lines, right Y-axis) or B) Children’s Hospital of Philadelphia Infant Test of Neuromuscular Disorders (CHOP-INTEND) scores (dashed lines, right Y-axis) over three timepoints: baseline, 6 months, and 14 months. Each color represents a different patient with Spinal Muscular Atrophy. For GFAP quantification, 5 subjects were evaluated at baseline, 5 at 6 months, and 6 at 14 months. For HINE, 9 subjects were evaluated at baseline, 6 at 6 months, and 9 at 14 months. For CHOP-INTEND, 9 subjects were evaluated at baseline, 6 at 6 months, and 9 at 14 months. Missing data points were not interpolated. Missing data related to GFAP quantification were due to the absence of biological samples at the corresponding timepoints, whereas missing clinical scale assessments occurred because evaluations at the time of interest were not performed, partly due to participant recruitment and follow-up challenges during the COVID-19 pandemic.
