# Supplementary material for: Unbiased bioinformatic and clinical analysis identifies glial fibrillary acidic protein as a potential biomarker in spinal muscular atrophy
Source: Brain Commun. 2026 Sep 11;8(5):fcag350. doi: 10.1093/braincomms/fcag350 (PMC13613212; doi:10.1093/braincomms/fcag350)
Supplement: Supplementary file 1 [file fcag350_supplementary_data.zip › Supplementary_Table_4.docx]

| **Supplementary Table 4. Correlations of serum NfL and GFAP levels with clinical characteristics and outcome measures** | | | | | | | | | |
| --- | --- | --- | --- | --- | --- | --- | --- | --- | --- |
|  | | **NFL baseline** | **GFAP baseline** | **Disease Duration** | **Age at Onset** | **HINE** | **ChopINTEND** | **HFMSE** | **RULM** |
| **NFL baseline** | Spearman's rho | 1,000 | ,375^**^ | -,491^*^ | -,754^**^ | -,236 | -,028 | -,012 | -,257 |
|  | Sig. (2-tailed) | . | ,008 | ,013 | ,000 | ,397 | ,918 | ,978 | ,623 |
|  | N | 49 | 49 | 25 | 25 | 15 | 16 | 8 | 6 |
| **GFAP baseline** | Spearman's rho | ,375^**^ | 1,000 | -,690^**^ | -,598^**^ | ,169 | ,236 | ,299 | -,029 |
|  | Sig. (2-tailed) | ,008 | . | ,000 | ,002 | ,548 | ,379 | ,471 | ,957 |
|  | N | 49 | 49 | 25 | 25 | 15 | 16 | 8 | 6 |
| **Disease Duration** | Spearman's rho | -,491^*^ | -,690^**^ | 1,000 | ,652^**^ | -,275 | -,448 | -,359 | -,657 |
|  | Sig. (2-tailed) | ,013 | ,000 | . | ,000 | ,320 | ,082 | ,382 | ,156 |
|  | N | 25 | 25 | 25 | 25 | 15 | 16 | 8 | 6 |
| **Age at Onset** | Spearman's rho | -,754^**^ | -,598^**^ | ,652^**^ | 1,000 | ,508 | ,345 | -,030 | ,543 |
|  | Sig. (2-tailed) | ,000 | ,002 | ,000 | . | ,053 | ,190 | ,944 | ,266 |
|  | N | 25 | 25 | 25 | 25 | 15 | 16 | 8 | 6 |
| **HINE** | Spearman's rho | -,236 | ,169 | -,275 | ,508 | 1,000 | ,935^**^ | 1,000^**^ | . |
|  | Sig. (2-tailed) | ,397 | ,548 | ,320 | ,053 | . | ,000 | . | . |
|  | N | 15 | 15 | 15 | 15 | 15 | 15 | 3 | 0 |
| **ChopINTEND** | Spearman's rho | -,028 | ,236 | -,448 | ,345 | ,935^**^ | 1,000 | 1,000^**^ | . |
|  | Sig. (2-tailed) | ,918 | ,379 | ,082 | ,190 | ,000 | . | . | . |
|  | N | 16 | 16 | 16 | 16 | 15 | 16 | 3 | 1 |
| **HFMSE** | Spearman's rho | -,012 | ,299 | -,359 | -,030 | 1,000^**^ | 1,000^**^ | 1,000 | ,800 |
|  | Sig. (2-tailed) | ,978 | ,471 | ,382 | ,944 | . | . | . | ,200 |
|  | N | 8 | 8 | 8 | 8 | 3 | 3 | 8 | 4 |
| **RULM** | Spearman's rho | -,257 | -,029 | -,657 | ,543 | . | . | ,800 | 1,000 |
|  | Sig. (2-tailed) | ,623 | ,957 | ,156 | ,266 | . | . | ,200 | . |
|  | N | 6 | 6 | 6 | 6 | 0 | 1 | 4 | 6 |
